# Analyzing Efficiency of Perovskite Solar Cells Under High Illumination Intensities by SCAPS Device Simulation

**DOI:** 10.3390/nano15040286

**Published:** 2025-02-13

**Authors:** Heng Li, Yongtao Huang, Muyan Zhu, Pingyuan Yan, Chuanxiang Sheng

**Affiliations:** 1 School of Microelectronics, Nanjing University of Science and Technology, Nanjing 210094, China; js_ypy@126.com; 2 School of Electronic and Optical Engineering, Nanjing University of Science and Technology, Nanjing 210094, China; hyt@njust.edu.cn (Y.H.); zhumuyan@njust.edu.cn (M.Z.); 3 School of Information Science and Technology, Fudan University, Shanghai 200433, China

**Keywords:** perovskite of CH_3_NH_3_PbI_3_, SCAPS, concentrating photovoltaics

## Abstract

The perovskite solar cell (PSC) is undergoing intense study to meet sustainable energy and environmental demands. However, large-sized solar cells will degrade the power conversion efficiency, thus concentrating light on small-size devices would be a solution. Here, we report the performance of a p–i–n structured device using CH_3_NH_3_PbI_3_ (MAPbI_3_) as the active layer with an area of 6 mm^2^. We prove that the power output would be up to 4.2 mW under 10 Suns compared to the 0.9 mW obtained under 1 Sun; however, this results in an actual efficiency drop of the PSC. Further, using a SCAPS device simulation, we found that the intrinsic properties, such as mobility and defect density, of MAPbI_3_ has no profound influence on the relationship between light intensity and power conversion efficiency (PCE), but the series resistance is the dominant limiting factor on the performance of the PSC under high illumination intensities. Our work suggests the potential of perovskite in concentrating photovoltaics and makes recommendations for future development.

## 1. Introduction

Since its emergence over 15 years ago, the organic–inorganic hybrid perovskite solar cells (PSCs) have achieved a power conversion efficiency (PCE) of over 26% [1,2,3]. At the same time, more possibilities about the application of PSCs are being explored, such as building-integrated photovoltaics, and even wallpapers [4,5]. Nevertheless, although much progress has been achieved to push the frontier of device fabrication [6], one of the biggest challenges for commercializing PSCs is the fabrication of large area devices. Nowadays, increasing the area to the range of ~cm^2^ could push the PCE over 20%, but the PCE drops rapidly as the active area is enlarged compared to the Silicon solar cell [7,8]. On the other hand, light controlling techniques, such as concentrating photovoltaics, may address the scalability problem [9]. However, less effort has been made on concentrating photovoltaics with perovskite so far [10,11]. Baig et al. investigated its possible application in a real working environment with an optical concentrator of a kaleidoscope with a truncated pyramid geometry. They also achieved a slight increase in PCE, namely 21.6%, under 1.78 Suns compared to the 21% under 1 Sun; then, the PCE decreases at about 10.7 Suns [12]. Wang et al. worked on concentrating photovoltaics based on various perovskites, in which the FA_0.83_Cs_0.17_PbI_2.7_Br_0.3_-based cell shows good performance at a higher irradiance level than for MAPbI_3_ [13].

In the current work, we fabricated a PSC with an active area of 6 mm^2^ based on CH3NH3PbI3 (MAPbI3). Using a continuous wave laser at 532 nm as an excitation source, we found that PCE decreases with intensity increases from 10 to 1000 mW/cm^2^, in general. Furthermore, with a SCAPS device simulation, we found that the relatively small mobility, as well as the existence of defects, are not the reasons for the attenuation of the PCE at high excitation intensities. Instead, the large series resistance (~few Ω·cm^2^) is the main reason. Since the series resistance could be intrinsically small by optimizing the contact resistance and electric properties of charge transfer layers, it will be possible to achieve a perovskite solar cell for concentrating photovoltaics

## 2. Experiments and Methods

PbI_2_ (>99.9%) and CH3NH3I (MAI) (>99.9%) were purchased from Xi’an Polymer Light Technology Corp. All raw materials and reagents were stored in a glove box under a nitrogen atmosphere and would be used directly without further purification if not specified.

The substrates of ITO-coated glass were cleaned consecutively in detergent, acetone, isopropanol, and ethanol ultrasonic baths for 15 min, respectively. A thin layer of PEDOT:PSS (J&K, 99.9%) was spin-coated onto the cleaned ITO at 5000 rpm for 50 s and then annealed at 140 °C for 20 min under ambient conditions. Then, the substrates were transferred into an N_2_ glove box for further device fabrication. MAI and PbI_2_ with a molar ratio of 1: 1 were dissolved in a DMF (Aladdin, 99.9%) and DMSO (Aladdin, 99.9%) mixed solvent (9: 1) with a concentration of 0.6 M (based on PbI_2_). The solutions were stirred overnight in glove box at ~50 °C and cooled to room temperature. The fresh precursor solution was spin-coated at 4000 rpm for 30 s onto the PEDOT:PSS-coated ITO substrates, 300 μL chlorobenzene was dropped onto the substrate at ~5 s, and then annealed at 100 °C for 20 min in a N_2_ glove box. PC_61_BM (Polymer Light, 99.9%) was dissolved in chlorobenzene (CB, Aladdin, 99.9%) with 20 mg/mL, and was subsequently deposited at 2000 rpm for 45 s. BCP (J&K, 99.9%) was dissolved in isopropanol (IPA) with 0.5 mg/mL, and was deposited at 4000 rpm for 30 s. A layer of silver (~100 nm) was deposited on the BCP layer through a shadow mask under vacuum (ca. 10^−4^ Pa). The effective area for the perovskite solar cell was 6 mm^2^. The simulations were performed by SCAPS, which is a well-known one-dimensional simulator based on solving continuity and Poisson equations [14,15].

## 3. Results and Discussion

### 3.1. Device Performance

**Figure 1:**
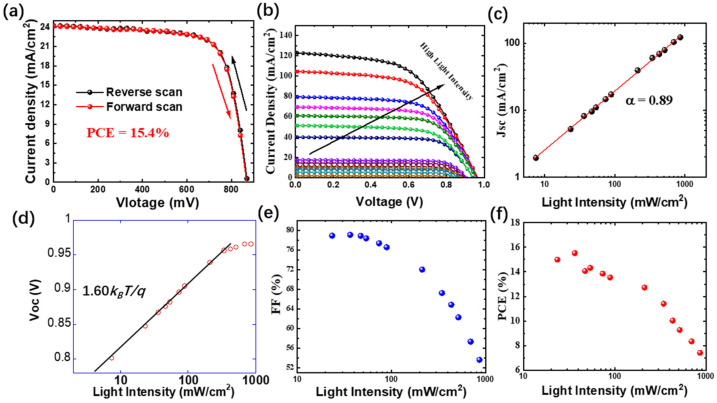
(**a**) Typical J-V curve of a MAPbI3 perovskite solar cell with the forward and reverse scan direction under a simulated AM 1.5 G illumination. (**b**) J-V curves of a MAPbI_3_ perovskite solar cell excited using a continuous wave laser of 532 nm at various intensities. (**c**–**f**): J_sc_, V_oc_, FF number, and PCE of a solar cell as the function of the intensity of the excitation laser.

In Figure 1, the typical experimental J-V curve of the PSC device was presented without hysteresis. The J-V measurements were conducted using a Keithley 2400 source meter under a simulated AM 1.5 G illumination (100 mW/cm^2^; Solarbeam-02-3A Class AAA Solar Simulator). In Figure 1b, using laser at 532 nm as an illumination source, the J-V curves at various excitation intensities are included. Figure 1c,d show the short-circuit current (J_sc_) and voltage of open circuit (V_oc_) as the function of excitation intensities, respectively. J_sc_ follows the power law dependence of light intensity, J_sc_ ∝ P_in_^α^, where α is a power law exponent and P_in_ is the laser intensity. α < 1 suggests that the trap-assisted recombination is a limiting factor for the current [16,17]. In Figure 1d of V_oc_ as the function of the illumination intensity, V_oc_ may follow the power law dependence on light intensity too, i.e., V_oc_ ∝ βk_B_T/q ln(P_in_), where k_B_ is the Boltzmann constant, P_in_ is the light intensity, and β is the ideality factor (quality factor). β = 1 and β = 2 stand for pure bimolecular recombination and trap-assisted recombination, respectively. Normally, β is between 1 and 2 for working devices [18,19]. At high intensities, V_oc_ becomes saturated with the light intensities, which may be caused by interfacial defects and non-radiative recombination losses in PSCs [20]. Figure 1e is the fill factor as the function of light intensity, which remains almost constant up to 60 mW/cm^2^, but decreases when the intensity increases [21]. Eventually, compared to Figure 1a, considering the active area (6 mm^2^) of the device, the power output was boosted up to ~4.2 mW under 10 Suns compared to 0.92 mW obtained under 1 Sun; on the other hand, it is obvious that the actual power transfer efficiency drops at higher illumination intensities. Thus, the current device may not be ideal for concentrating photovoltaics.

### 3.2. Simulation Results

**Figure 2:**
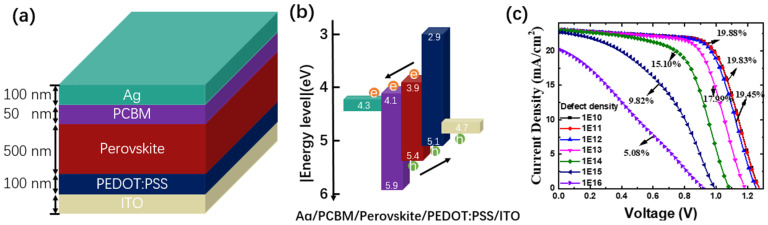
(**a**) Device structure diagram. (**b**) Device structure and energy band diagram of each layer. (**c**) Simulated J-V curves at various defect intensities.

To understand why the PSC here is not ideal for concentrating photovoltaic applications, we tried to simulate concentrating photovoltaics using SCAPS. The device structure used in the simulation is shown in Figure 2a, which is ITO-glass/PEDOT:PSS/CH_3_NH_3_PbI_3_/PCBM/Ag, and the thickness of each layer is 1 mm/100 nm/500 nm/50 nm/100 nm, respectively. The energy diagram is shown in Figure S1 of Supplemental Materials. The HOMO energy level of PCBM is 5.9 eV and the LUMO energy level of PEDOT:PSS is 2.9 eV, forming a hole blocking layer and an electron blocking layer, respectively. Thus, the p–i–n structure of the solar cell is formed. Because the exciton binding energy in MAPbI_3_ is about 10–20 meV, the free carriers can be photo-generated directly after illumination at room temperature [22,23]. The working temperature is set as 300 K, and the absorption spectra was simulated using the following equation [24]:

(1) $$\alpha \left(E\right)=\left(\alpha_{0}+\beta_{0}\frac{E_{g}}{hv}\right)\sqrt{\frac{hv}{E_{g}}-1}$$

where *E_g_* is the bandgap (1.5 eV), *hν* is the photon energy, and *α*_0_ and *β*_0_ are constants (*α*_0_ = 10^5^ cm^−1^; *β*_0_ = 10^−12^ cm^−1^) [24].

The parameters used in SCAPS are summarized in Table 1, in which the trap density (simplified at energy level 0.4 eV above the valence band) was used as an adjusting parameter. The thickness dependent device performance is also included in Figure S2 of Supplemental Materials, setting the perovskite layer as 500 nm. We increased the defect density from 1 × 10^10^ cm^−3^ to 1× 10^16^ cm^−3^. The simulated J-V curve is shown in Figure 2c. Although the 1 × 10^10^ cm^−3^ and 1 × 10^11^ cm^−3^ are almost identical, the increase in defect density reduces the efficiency of the device from the initial 19.88% (1 × 10^10^/cm^3^) to 5.08% (1 × 10^16^/cm^3^), and the V_oc_, J_sc_, and particularly the fill factor, also decrease as the trap density increases. We may conclude that, although perovskites are recognized to have a considerable defect tolerance [25], defect density is obviously still an important factor in determining the performance of the devices.

**Figure 3:**
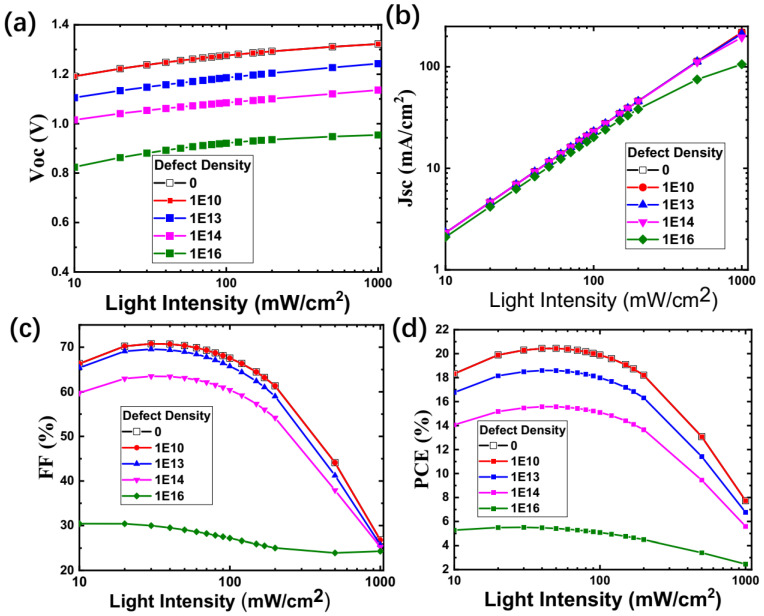
(**a**–**d**): V_oc_, J_sc_, FF number, and PCE as a function of light intensities at various defect densities.

In Figure 3a, the simulated V_oc_ as the function of light intensity is shown, and we found that although V_oc_ values decrease with the defect density increases at the same excitation intensity, the relationship between light intensity and V_oc_ are basically the same, as shown by the J_sc_ in Figure 3b. The FF number, which decreases, in general, with the light intensity, increases for all defect intensities. At last, the PCE increases slightly from 10 mW/cm^2^ to ~around 50 mW/cm^2^ and decreases from 50 mW/cm^2^ to 1000 mW/cm^2^. Again, the behavior between PCE and excitation intensity are similar at various defect intensities, i.e., a higher defect intensity results in a lower PCE value.

On the other hand, solar cells based on GaAs are a well-known example for concentrating photovoltaics. Compared with perovskite and MAPbI_3_, GaAs’s electron (hole) effective mass is calculated as 0.067 (0.45), respectively [38], while MAPbI_3_’s electron (hole) effective mass [34] is calculated as 0.11 (0.21), respectively and they are close to each other. GaAs and MAPbI_3_ share a similar bandgap. Initially, perovskite was regarded as the analog of GaAs for solar cell applications. However, one of the biggest differences between perovskite and GaAs is in their mobility. In GaAs, the mobility of the electron (hole) could be as high as 8500 cm^2^V^−1^s^−1^ (400 cm^2^V^−1^s^−1^) [39].

**Figure 4:**
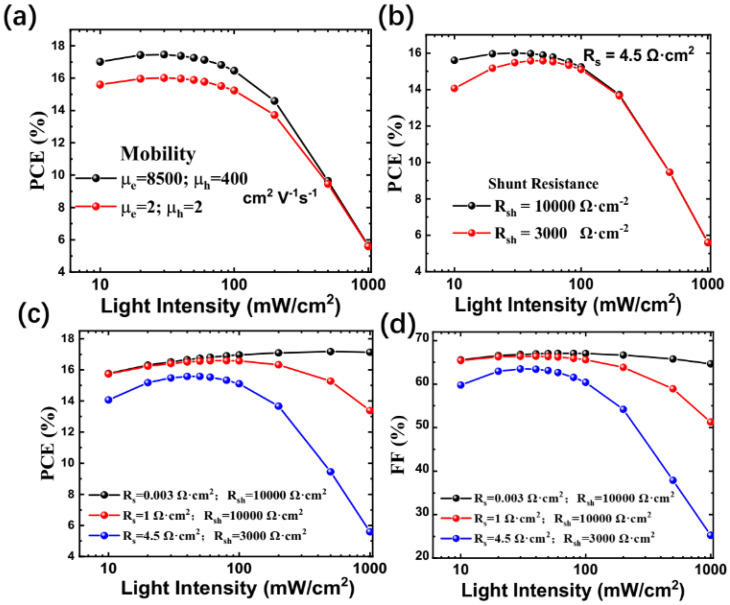
(**a**) Simulated PCE as the function of light intensity at two groups of electron and hole mobility. (**b**) Simulated PCE as the function of light intensity at two shunt resistances, and the series resistance is fixed at 4.5 Ω·cm^2^. (**c**,**d**): Simulated PCE and FF number at three groups of R_s_ and R_sh_.

Thus, in simulation, after assuming that the mobility of carriers in perovskite would be same as with GaAs, we compared the simulated results with high and low mobilities, as shown in Figure 4a. Here, the defect concentration was set at 1 × 10^14^ [40] because the simulated results shown in Figure 2c with a defect density of 1 × 10^14^ is close to the experimental results shown in Figure 1a. Nevertheless, we found, in the current work, that mobility does not influence PCE too much. More importantly, mobility does not influence the relationship between PCE and light intensity. In fact, at a higher excitation intensity at 1000 mW/cm^2^, the mobility does not influence PCE at all. Thus, a small mobility may not be a major hindrance for concentrating photovoltaics of perovskites.

Since the intrinsic properties of perovskite such as its mobility and defect concentrations have no profound influence on the relationship between PCE and light intensity, we focus on two other parameters for devices, namely series resistance (R_s_) and shunt resistance (R_sh_). Firstly, the series resistance was set as 4.5 Ω·cm^2^, which is the same as the value shown in Table 1. We compared the two shunt resistances, 3000 Ω·cm^2^ and 10,000 Ω·cm^2^, and found that the values of R_sh_ do not influence the relationship between PCE and light intensity. Furthermore, we experimented with a small value of R_s_ as shown in Figure 4c (PCE) and Figure 4d (FF number), respectively. The black dotted line, red dotted line, and blue dotted line, respectively, represent 0.003 Ω·cm^2^ (R_s_) + 10,000 Ω·cm^2^ (R_sh_), 1 Ω·cm^2^ (R_s_) + 10,000 Ω·cm^2^ (R_sh_), and 4.5 Ω·cm^2^ (R_s_) + 3000 Ω·cm^2^ (R_sh_), respectively. The black dotted line is the optimal combination of low series resistance and high shunt resistance, which are similar to GaAs solar cells [41]. The red line represents a higher R_s_ and high R_sh_, which represents the reported MAPbI_3_ perovskite solar cells [37], while the blue dotted line represents a high R_s_ and low R_sh_, which is the same as the experiment shown in Figure 1. These prove that series resistance is the main reason for the decrease in FF number and PCE under high illumination intensities. In the current perovskite solar cells, which is considered to be a p–i–n structure, its series resistance could be determined by the resistances of the hole transport layer and the electron transport layer, as well as the resistance of the metal contacts to these layers. The main impact of series resistance is on reducing the fill factor at high illumination intensities, as shown in Figure 4d, although excessively high values may also reduce the short-circuit current. Nevertheless, the concentrated solar illumination may cause a higher working temperature, negatively impacting the performance and stability. The effect of high illumination may also change the charge carrier mobility and carrier lifetime in the working materials of solar cells. These problems would have to be successfully addressed before the application of perovskite-based concentrator photovoltaics.

In conclusion, experimentally, we found that the solar cell used in the current work delivers high electric power under higher illumination intensities; however, the PCE is relatively smaller, thus being not ideal for concentrating photovoltaics. Using a SCAPS simulation, we found that this is not due to the intrinsic properties of perovskite film such as low mobility as well as defect intensity; however, the large series resistance (R_s_) is the main reason. Since R_s_ is mainly due to properties such as the contact resistance between the interface of metal and charge transfer layers, our work suggests that optimizing charge transfer layers and contact metals to reduce the device series resistance would be beneficial for the further development of concentrating photovoltaics based on perovskites.

## Figures and Tables

**Table 1:** Parameters used for simulation in SCAPS.

	PCBM	MAPbI_3_	PEDOT:PSS
Thickness (nm)	50	500	100
Bandgap (eV)	1.8 [26,27]	1.5 [28]	2.2 [29]
Electron affinity (eV)	4.1 [26]	3.9 [28]	2.9 [29]
Relative dielectric constant	3.9 [30]	32 [31]	3 [29]
Effective state density of conduction band (cm^−3^)	2.5 × 10^21^ [30]	2.49 × 10^18^ [32]	1 × 10^19^ [29]
Effective state density of valance band (cm^−3^)	2.5 × 10^21^ [30]	6.98 × 10^18^ [32]	1 × 10^19^ [33]
Hole mobility (cm^2^/V·s)	2 × 10^−1^ [30]	2 [34]	0.01 [34]
Electron mobility (cm^2^/V·s)	2 × 10^−1^ [30]	2 [34]	7.7 × 10^−1^ [35]
Defect type	Neutral	Neutral	Neutral
Defect density (cm^−3^)	1 × 10^15^ [30]	**	3.7 × 10^17^ [36]
Series resistance (Ω·cm^2^)		4.5 [37]	
Shunt resistance (Ω·cm^2^)		3000 [37]	

** adjustable parameter.

## Data Availability

The data will be made available upon request.

## Supplementary Materials

The following supporting information can be downloaded at: https://www.mdpi.com/article/10.3390/nano15040286/s1.
