# Supplementary material for: Analyzing Efficiency of Perovskite Solar Cells Under High Illumination Intensities by SCAPS Device Simulation
Source: Nanomaterials (Basel). 2025 Feb 13;15(4):286. doi: 10.3390/nano15040286 (PMC11858504; doi:10.3390/nano15040286)
Supplement: Supplementary file 1 [file nanomaterials-15-00286-s001.zip › nanomaterials-3399937-supplementary.pdf]

Supplementary Materials

Analyzing Efficiency of Perovskite Solar Cells Under High Illumination Intensities by SCAPS Device Simulation

Heng Li [1,*], Yongtao Huang [2], Muyan Zhu [2], Pingyuan Yan [1], Chuanxiang Sheng [2,3,*]

[1] School of Microelectronics, Nanjing University of Science and Technology, Nanjing 210094, China; js_ypy@126.com
[2] School of Electronic and Optical Engineering, Nanjing University of Science and Technology, Nanjing 210094, China; hyt@njust.edu.cn (Y.H.); zhumuyan@njust.edu.cn (M.Z.)
[3] School of Information Science and Technology, Fudan University, Shanghai 200433, China
* Correspondence: hengli@njust.edu.cn (H.L.); cxsheng@fudan.edu.cn (C.S.)

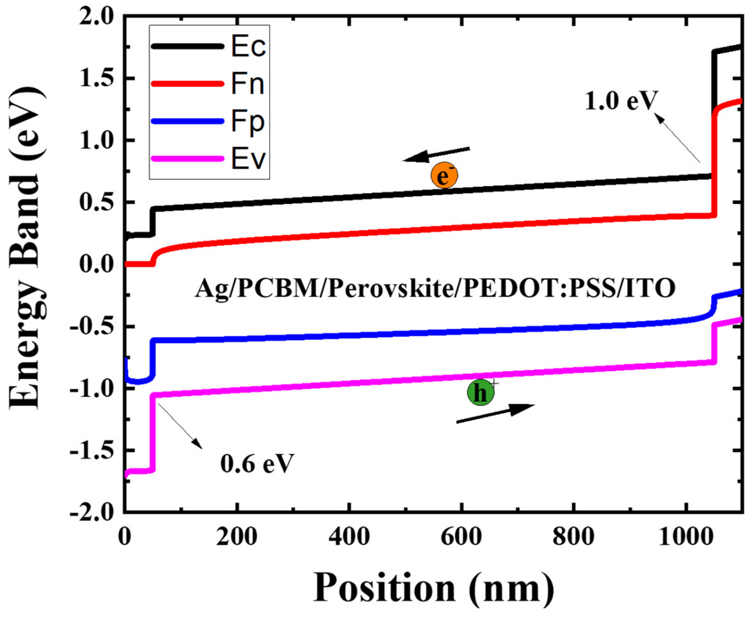

Figure S1. typical energy diagram shown in SCAPS-1D.

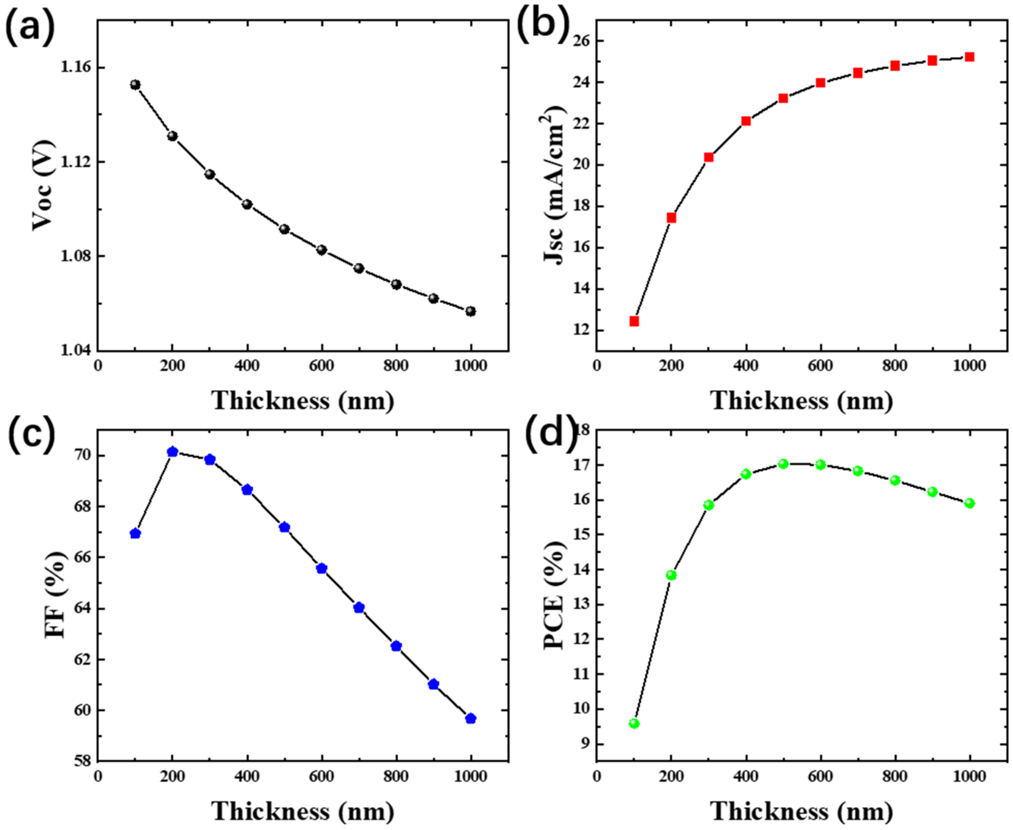

Figure S2. Perovskite thickness dependence of device performance. (**a**) Voc (**b**) Jsc (**c**) FF number (**d**) power conversion efficiency (PCE).

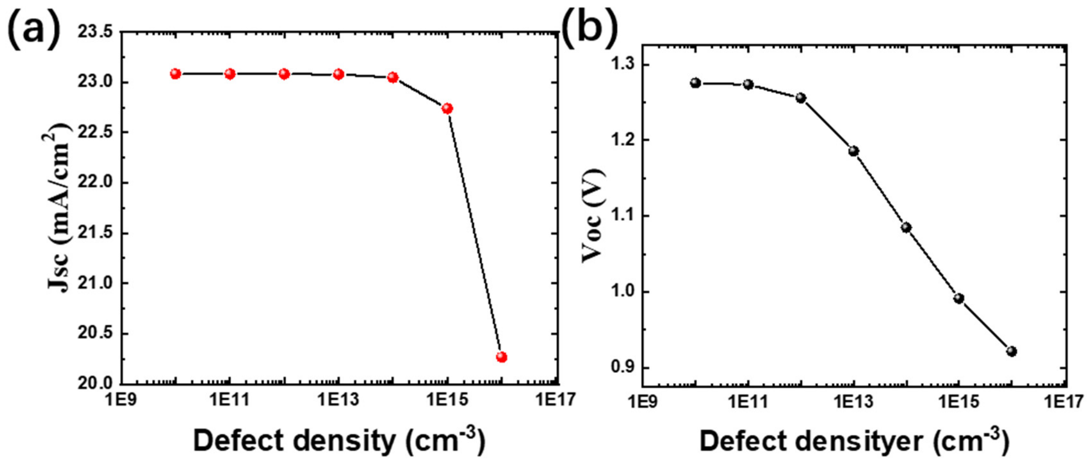

Figure S3. defect density dependence of Jsc and Voc for simulated PCE of Figure 2c.

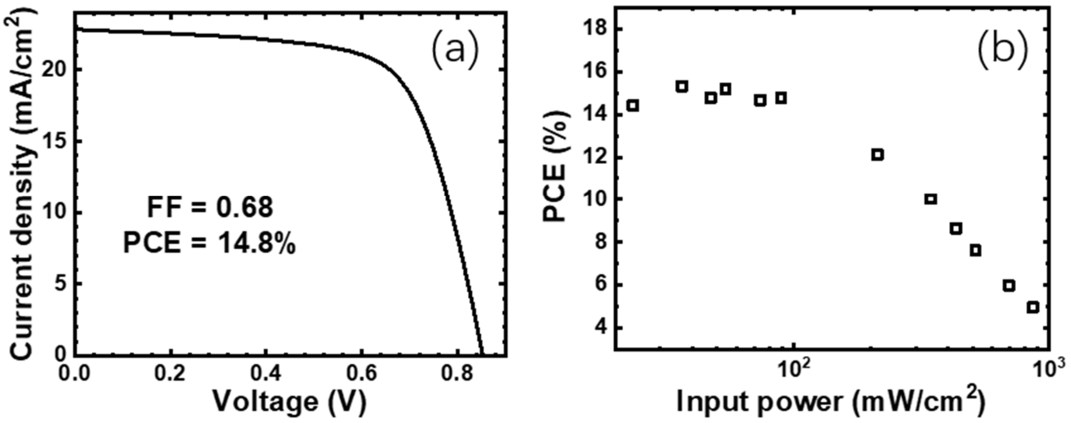

Figure S4. The experimental results of another device based on MAPbI3. (**a**) a typical J-V curve. (**b**) PCE as a function of illumination intensity.
